# Contribution of Multiparameter Flow Cytometry Immunophenotyping to the Diagnostic Screening and Classification of Pediatric Cancer

**DOI:** 10.1371/journal.pone.0055534

**Published:** 2013-03-05

**Authors:** Cristiane S. Ferreira-Facio, Cristiane Milito, Vitor Botafogo, Marcela Fontana, Leandro S. Thiago, Elen Oliveira, Ariovaldo S. da Rocha-Filho, Fernando Werneck, Danielle N. Forny, Samuel Dekermacher, Ana Paula de Azambuja, Sima Esther Ferman, Paulo Antônio Silvestre de Faria, Marcelo G. P. Land, Alberto Orfao, Elaine S. Costa

**Affiliations:** 1 Pediatric Institute IPPMG, Universidade Federal do Rio de Janeiro (UFRJ), Rio de Janeiro, Brazil; 2 Department of Pathology, Faculty of Medicine, UFRJ, Rio de Janeiro, Brazil; 3 Servidores do Estado Hospital (HSE), Rio de Janeiro, Brazil; 4 Pediatric Hematology and Oncology Program, Cancer Research Center, Brazilian National Cancer Institute (INCa), Rio de Janiero, Brazil; 5 Clinic Hospital, Federal University of Paraná (UFPR), Curitiba, Brazil; 6 Department of Pediatric Oncology/Brazilian National Cancer Institute (INCa), Rio de Janiero, Brazil; 7 Cytometry Service, Department of Medicine and Cancer Research Center (IBMCC, University of Salamanca-CSIC and IBSAL), University of Salamanca, Salamanca, Spain; Health Canada, Canada

## Abstract

Pediatric cancer is a relatively rare and heterogeneous group of hematological and non-hematological malignancies which require multiple procedures for its diagnostic screening and classification. Until now, flow cytometry (FC) has not been systematically applied to the diagnostic work-up of such malignancies, particularly for solid tumors. Here we evaluated a FC panel of markers for the diagnostic screening of pediatric cancer and further classification of pediatric solid tumors. The proposed strategy aims at the differential diagnosis between tumoral *vs*. reactive samples, and hematological *vs*. non-hematological malignancies, and the subclassification of solid tumors. In total, 52 samples from 40 patients suspicious of containing tumor cells were analyzed by FC in parallel to conventional diagnostic procedures. The overall concordance rate between both approaches was of 96% (50/52 diagnostic samples), with 100% agreement for all reactive/inflammatory and non-infiltrated samples as well as for those corresponding to solid tumors (n = 35), with only two false negative cases diagnosed with Hodgkin lymphoma and anaplastic lymphoma, respectively. Moreover, clear discrimination between samples infiltrated by hematopoietic *vs.* non-hematopoietic tumor cells was systematically achieved. Distinct subtypes of solid tumors showed different protein expression profiles, allowing for the differential diagnosis of neuroblastoma (CD56^hi^/GD2^+^/CD81^hi^), primitive neuroectodermal tumors (CD271^hi^/CD99^+^), Wilms tumors (>1 cell population), rhabdomyosarcoma (_nu_MYOD1^+^/_nu_myogenin^+^), carcinomas (CD45^−^/EpCAM^+^), germ cell tumors (CD56^+^/CD45^−^/NG2^+^/CD10^+^) and eventually also hemangiopericytomas (CD45^−^/CD34^+^). In summary, our results show that multiparameter FC provides fast and useful complementary data to routine histopathology for the diagnostic screening and classification of pediatric cancer.

## Introduction

More than 200,000 pediatric patients (<15 years old) are diagnosed with cancer every year [1]. Despite cancer is the leading cause of disease-related death in children in most countries [2], individual outcomes largely depend on fast tumor diagnosis, classification and staging, for appropriate therapy selection and maximized cure rates [3]. Since a significant proportion of pediatric tumors lack morphological evidence of differentiation and histological origin, a battery of immunocytochemical markers and in situ hybridization, ultrastructural and molecular diagnostic procedures, are typically required for the diagnostic classification of the disease [3]–[7].

For several decades, multiparameter flow cytometry (MFC) immunophenotyping has proven to be essential for rapid diagnosis, classification and monitoring of therapy of most hematological malignancies, including pediatric leukemias and lymphomas. Conversely, it remains a research tool for pediatric solid tumors [8]–[15]. Such limited usage of MFC in the diagnosis of pediatric solid tumors probably relates to the need for single cell suspensions and the availability of relatively restricted panels of reliable and validated markers, among other factors [16]–[18]. Thus, usage of flow cytometry in pediatric solid tumors has been almost restricted to the evaluation of tumor cell DNA contents in both paraffin-embedded and frozen tissue specimens, in combination or not with simultaneous staining for one or a few phenotypic markers [19]–[24]. Consequently, while immunophenotypic studies are routinely applied in most pediatric lymphomas for the differential diagnosis between B- and T-cell precursor lymphoblastic lymphomas and Burkitt lymphoma 25–28, few studies have been reported so far, in which MFC is systematically applied to the study of the phenotypic characteristics of pediatric solid tumors [29]–[31]. In addition, the few reported studies have mainly focused on descriptive analyses of the staining patterns for one or a few markers, usually in a single diagnostic subtype of the disease.

Despite all the above, preliminary studies have shown that neuroendocrine tumors display a CD45^−^/CD56^+^ phenotype [32]–[35]; in turn, tumor cells from neuroblastoma coexpress GD2 [35], whereas primitive neuroectodermal tumors (PNET) coexpress CD57 and CD99, as well as CD56 [31], [34], [36]–[38], and rhabdomyosarcoma tumor cells are myogenin^+^ [31], [39]–[41]. Despite this, the specificity of these phenotypes among the distinct subtypes of pediatric solid tumors remains to be established, and no study has been reported so far, in which a comprehensive panel of markers aimed at diagnostic screening, differential diagnosis and classification of distinct types of pediatric tumors, has been proposed and evaluated.

Here we evaluated a new comprehensive MFC panel of markers for the diagnostic screening of pediatric cancer and correct assignment of pediatric solid tumors to specific disease entities.

## Materials and Methods

### Patients and Samples

A total of 52 samples from 40 patients suspicious of pediatric cancer –21 males (52.5%) and 19 females (47.5%) - were collected between November 2009 and December 2011, at three distinct centers: Instituto de Puericultura e Pediatria Martagão Gesteira/Universidade Federal do Rio de Janeiro (IPPMG/UFRJ) and Hospital Servidores do Estado (HSE), both from Rio de Janeiro (Brazil), and Hospital de Clínicas/Universidade Federal do Paraná (HC/UFPR) in Curitiba (Brazil). Median patient age was of 5 years (range: 1–14 years). Most samples (48/52; 92.3%) were analyzed at diagnosis.

In all cases, final diagnosis and classification was established based on morphological and immunohistochemical analyses performed at a reference laboratory, following the World Health Organization (WHO) criteria [42]–[44] Thirty-one patients (78%) had cancer, 17 of whom (55%) showed metastatic disease; the remaining 9 children (23%) had inflammatory/reactive diseases. According to histopathological/immunohistochemical diagnosis, 11 patients (12 samples) had neuroblastoma, 3 (4 samples) had rhabdomyosarcoma, 2 (2 samples) a primitive neuroectodermal tumor (PNET), 8 (9 samples) had lymphoma, 2 (2 samples) Wilms tumors, 2 (3 samples) germ cell tumors, and one patient had an adrenal carcinoma, one a nasopharyngeal carcinoma, and one an hemangioperycitoma; the other 17 specimens corresponded to 9 reactive samples and 8 samples from patients with neoplastic diseases which showed no tumor infiltration. The specific origin of the specimens is detailed in Table S1.

### Ethics Statement

The study was approved by the Ethics Committee of the IPPMG and HSE hospitals and samples were obtained after informed consent was given by the children and their parents or guardians, according to the Helsinki Declaration protocol. Parents/guardians provided their written informed consent to participate in this study and this written informed consent was approved by the local ethics committees.

### Preparation of Tissue Samples and Body Fluids

Tissue samples free from fat and necrotic tissue (mean weight: 144 mg; range: <3 to 3,600 mg) were collected at the surgical unit. They were directly evaluated by an experienced pathologist, and divided into two contiguous blocks. One block was fixed in formalin, embedded in paraffin and processed for routine histopathology, while the other was placed in cold (4°C) phosphate-buffered saline (PBS; pH = 7.4) for further flow cytometric analyses. The later sample was weighted, placed in a Petri dish with PBS containing 1% bovine serum albumin (BSA; Calbiochem, La Jolla, CA), minced into small pieces (2–4 mm) with a scalpel blade and mechanically disaggregated with two sterile needles; afterward, it was filtered through a sterile Filcon syringe (100 µm pore size) to eliminate cell clumps and debris, centrifuged (10 min at 540 g) and resuspended in PBS containing 1% BSA at a final concentration of 10^6^ cells/mL. Afterward, the sample was immediately stained for MFC immunophenotyping. Bone marrow (BM) and other body fluid samples were stained either directly or after a centrifugation step (e.g. urine), as described below.

### Multiparameter Flow Cytometry Immunophenotypic Studies

**Figure 1:**
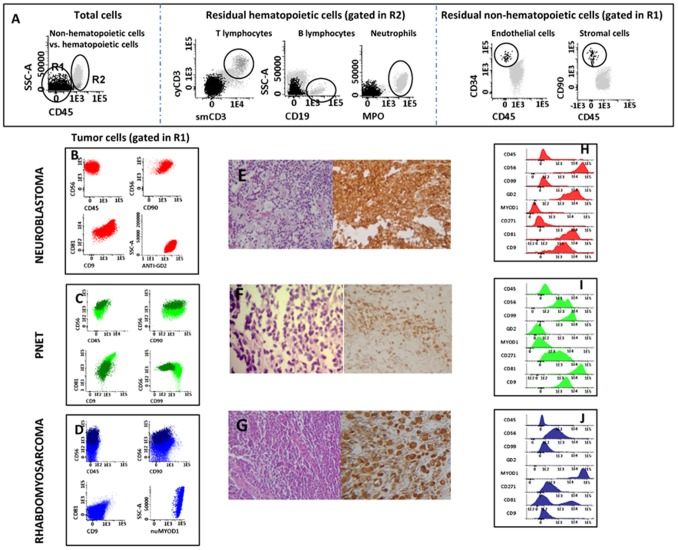
Immunophenotypic identification and chraracterization of pediatric tumor samples. In panel A, an illustrating example of the gating strategy and bivariate dot plot combinations used for the identification of CD45− tumor cells, CD45− residual stromal cells (e.g. endothelial cells and mesenquimal cells) and infiltrating hematopoietic cells (e.g. neutrophils, B and T cells) is shown. In turn, in panels B to J the immunophenotypic profile of CD45− tumor cells from a neuroblastoma (panels B and H), a PNET (panels C and I) and a rhabdomyossarcoma (panels D and J) tumor are shown together with representative pictures of the histophathological and immunohistochemical profiles of the same tumors stained with hematoxilin & eosin plus cromogranin (neuroblastoma cells in panel E), CD99 (PNET cells in panel F) and _(nu)_myogenin (rhabdomyossarcoma cells in panel G).

Each sample was stained with the following combinations of fluorochrome-conjugated monoclonal antibodies (MAbs) – fluorescein isothiocyanate (FITC)/phycoerythrin (PE)/PE-cyanin7(PECy7)/peridinin chlorophyll protein-Cy5.5 (PerCP-Cy5.5)/allophycocyanin (APC)/APC-Hilite7 (APC-H7)/pacific blue (PacB)/pacific orange (PacO) – devoted to simultaneous identification of tumor cells and normal/reactive B- and T-lymphocytes, monocytes and neutrophils :i) cytoplasmic (Cy) MPO/_Cy_CD79a/CD19/CD34/CD7/surface membrane (Sm) CD3/_Cy_CD3/CD45 [45]; ii) CD8-surface membrane immunoglobulin (_Sm_Ig)λ/CD56-sIgκ/−/CD19+CD4/CD3/−/CD20/CD45 (orientation panel in Table S2). Whenever suspicious tumor cells were detected following the gating strategy illustrated in figure 1, further phenotypic characterization of such suspicious tumor cells was performed with distinct characterization panels depending on the nature of the cells: non-hematopoietic vs B vs T vs other hematopoietic cells (Table S2). Staining of cells from solid tissues or BM, peripheral blood (PB) and other body fluids was performed as previously described in detail [46], [47]. Briefly, the samples (50 µl per tube) were incubated for 15 min at room temperature in the dark, in the presence of 3–20 µl of each of the above mentioned monoclonal antibodies (MAb), according to the recommendations of the manufacturers. Afterward, 2 ml of FACS lysing solution (Becton Dickinson) diluted 1∶10 (v/v) in distilled water was added, and the samples were incubated for another 10 min under the same conditions as those mentioned above, in order to lyse non-nucleated red cells. Then, cells were centrifuged (5 min at 540 g) and the cell pellet was washed twice with 4 ml of PBS. Finally, cells were resuspended in 0.5 ml of PBS. For the evaluation of cytoplasmatic (Cy) or nuclear (nu) antigens, cells were fixed, immediately after they were incubated with the MAb directed against cell surface membrane markers (see above); then, they were permeabilized and stained with MAb directed against the cytoplasmic and/or nuclear antigens, using the Fix & Perm reagent kit (Invitrogen, Carlsbad, CA, USA), strictly following the recommendations of the manufacturer. For unconjugated MAb, after incubation with the specific MAbs, a washing step followed by a second incubation step with a FITC-conjugated anti-mouse IgG reagent (Cytognos SL, Salamanca, Spain), was performed (15 min) in the dark. In order to confirm the specificity of the labelings, a negative control with an unlabeled sample was systematically run in parallel. Stained cells were acquired at low speed in a FACSCanto II flow cytometer –Becton/Dickinson Biosciences (BD), San José, CA, USA, - using the FACSDiVa software (BD). All except two samples, were processed within the first 4 h after surgery. For data analysis, the INFINICYT™ software program (Cytognos SL) was used. Antigen expression was used to identify the different types of cells present in the sample and each cell population identified was classified as being negative (−), dim positive (+lo), intermediate positive (+) and strongly positive (+hi) using arbitrary relative linear mean fluorescence intensity (MFI) values of 10^0^–10^2^, 10^2^–10^3^, 10^3^–10^4^, > 10^4^, respectively, depending on the MFI values observed vs baseline autofluorescence levels found in the control tube; antigen expression for a given marker was described as being heterogeneous, when variable expression levels were detected for that marker within a cell population.

Exclusion of non-viable tumor cells was based on their lower forward (FSC) and sideward (SSC) light scatter features; median cell viability of tissue specimens was of 75%. In 19/33 (57.5%) of solid tissue samples, parallel staining with propidium iodide (PI, Sigma,St Louis, MO, USA) was performed to evaluate cell viability, >90% of the PI-stained dead cells systematically corresponding to events which were excluded as non-viable cells in the FSC/SSC gate.

### Histological and Immunohistochemical Analyses

Histophatological examination was performed on hematoxilin/eosin (H&E) stained tissue sections (3 µm). In all samples where tumor infiltration was suspected and/or detected, tissue sections were also stained by conventional immunohistochemistry for the CD99, nuclear _(nu)_MYOD1, _(nu)_myogenin, synaptophysin, chromogranin, NSE, LCA, CD20, CD19, Ki67 and CD10 markers. Stained slides were independently assessed by two experienced pathologists. For BM, PB and urine samples, conventional cytological analysis was performed.

### Statistical Methods

In order to establish the statistical significance of differences observed between groups, the Mann-Whitney U test was used (continuous variables; SPSS software program, version 18.0, SPSS Inc., Chicago, IL, USA). The number of true-positive (TP; samples with the presence of a tumor cell population as detected by both conventional diagnostic techniques and flow cytometry), true-negative (TN; samples without tumor cell population as measured by conventional approaches and flow cytometry), false-positive (FP; samples with the presence of a tumor cell population by flow cytometry not detected by the reference methods) and false negative cases (FN; samples with undetectable tumor cells by flow cytometry but positive by the reference approaches), were calculated. Sensitivity and specificity were defined as TP/TP+FN and TN/TN+FP respectively, whereas the positive (PPV) and negative predictive values (NPV) were calculated as TP/TP+FP and TN/TN+FN, respectively.

## Results

### Differential Diagnosis between Reactive/Non-infiltrated and Tumor/infiltrated Samples

Based on the screening panel (panel 1 in Table S2), 9/52 samples (17%) corresponded to reactive samples; another 8 (15%) samples obtained from cancer patients for disease staging or monitoring purposes, were also negative for malignancy. All other samples (n = 35; 68%) showed infiltration by tumor cells. The overall concordance rate between MFC and conventional histopathological, immunohistochemical and/or cytological diagnostic procedures was of 96% (50/52 samples). In detail, a 100% agreement was attained for the 17 reactive/inflammatory and non-infiltrated samples, whereas a 94% concordance rate (33/35 samples) was achieved by MFC for infiltrated tumor samples. The two tumor samples which were misclassified by MFC corresponded to samples infiltrated by Hodgkin lymphoma cells and an anaplastic lymphoma, respectively. All samples infiltrated by solid tumors and B- or T-cell lymphomas were correctly identified (Table 1). Therefore, an overall efficiency of 96% (100% specificity and 94% sensitivity) was reached (PPV of 100% and NPV of 90%). The cellular composition of both reactive/inflammatory and other non-infiltrated samples is shown in Table S3, while the distribution of stromal cells (inflammatory, endothelial and fibroblasts/mesenchymal cells) in 14/26 samples that contained non-hematopoietic solid tumors is shown in Table S4.

**Table 1:** Comparison between multiparameter flow cytometry (MFC) and histopathology plus immunohistochemistry (IH) as regards identification of infiltration by neoplastic cells versus normal/reactive cells.

Diagnostic category	N. of concordant samples by MFC vs. IH/Total samples	(%)
**Non-infiltrated samples:**	17/17	100
Reactive/inflammatory samples	9/9	100
Other non-infiltrated samples	8/8	100
**Infiltrated samples:**	33/35	94
Lymphomas:	7/9	78
T-cell precursor LL	2/2	100
B-cell precursor LL	2/2	100
Diffuse large B cell lymphoma	1/1	100
Burkitt lymphoma	2/2	100
Hodgkin lymphoma	0/1	0
Anaplastic lymphoma	0/1	0
Solid Tumors:	26/26	100
Neuroblastoma	12/12	100
- Gaglioneuroblastoma	1/1	100
Rhabdomyosarcoma	4/4	100
Ewing Sarcoma/PNET	2/2	100
Adrenal carcinoma	1/1	100
Nasopharyngeal carcinoma	1/1	100
Hemagiopericytoma	1/1	100
Germ cell tumor	3/3	100
Wilmstumor	2/2	100
Total	50/52	96

All 8 samples were obtained from pediatric cancer patients, but they were all negative for the presence of tumor cells by conventional diagnostic approaches. LL: lymphoblastic lymphoma; PNET- primitive neuroectodermal tumor.

### Identification of Lymphoma *Versus* Non-hematopoietic Tumor Cells

A total of 35 samples (from 31 patients) contained tumor cells based on the screening panel (panel 1 in Table S2) and the characterization panels (panels 2 to 5 in Table S2). From them, 9 corresponded to malignant hematopoietic cells and 26 to non-hematopoietic solid tumors. In the two false-negative lymphoma cases, a rich polyclonal lymphocyte infiltrate was observed by MFC, without a clearly defined tumor cell population. In turn, all B-cell lymphoma cases (5/5) could be easily distinguished from pediatric solid tumors with screening panel 1 (Table S2) by the expression of B-cell markers (CD19^+^, cyCD79a^+^, CD22^+^) in association with CD45^+lo^ or CD45^+^, while these markers were systematically absent in all 26 pediatric solid tumors. With the B-cell characterization panel (panel 3 in Table S2) three B-cell lymphoma samples showed surface membrane _(Sm)_Ig light chain restriction (2 cases _Sm_Igλ^+^ and one _Sm_Igκ^+^) together with a Tdt^+^, CD20^+hi^, CD38^+hi^, CD10^+hi^, cyBcl2^−^ immunophenotype which is highly characteristic of childhood Burkitt lymphoma, except for TdT expression. The other two B-cell tumors presented with a CD45^+lo^, CD19^+^, CD20^−^, CD22^+^, CD10^−^, CD38^+^, CD58^+^, CD81^+^, CD9^+^, _Sm_Ig^−^, _Cy_Igµ^−^ phenotype compatible with B-cell precursor acute lymphoblastic lymphoma (BCP-ALL) by both MFC and histopathology. In turn, 2 T-cell lymphoma samples could be easily distinguished from both pediatric solid tumors and B-cell lymphomas with the screening panel (panel 1 in Table S2) based on their CD45^+lo^, _Sm_CD3^+lo^ and _Cy_CD3^+^ phenotype; in addition, these cells also showed a CD4^+^, CD8^−^, CD1a^+^, CD99^+^, CD2^+^, CD5^+^, CD27^+^, CD71^+^ and CD81^+^ phenotypic profile, lacking CD7^−^, CD117^−^ and B-cell markers once the appropriate screening and characterization panels (panels 1 and 4 in Table S2, respectively) were used. All 26 non-hematopoietic solid tumors analyzed were negative for all B and T-cell specific antigens investigated with the screening panel (panel 1 in Table S2), and they were CD45^−^. Twenty-two of these later cases (84%) expressed CD56^+^, together with variable patterns of expression of other markers associated with different non-hematopoietic tissues which were included in the characterization panel for solid tumors (panel 2 Table S2) as described below.

### Immunophenotypic Characterization of Non-Hematopoietic Tumor Cells

**Figure 2:**
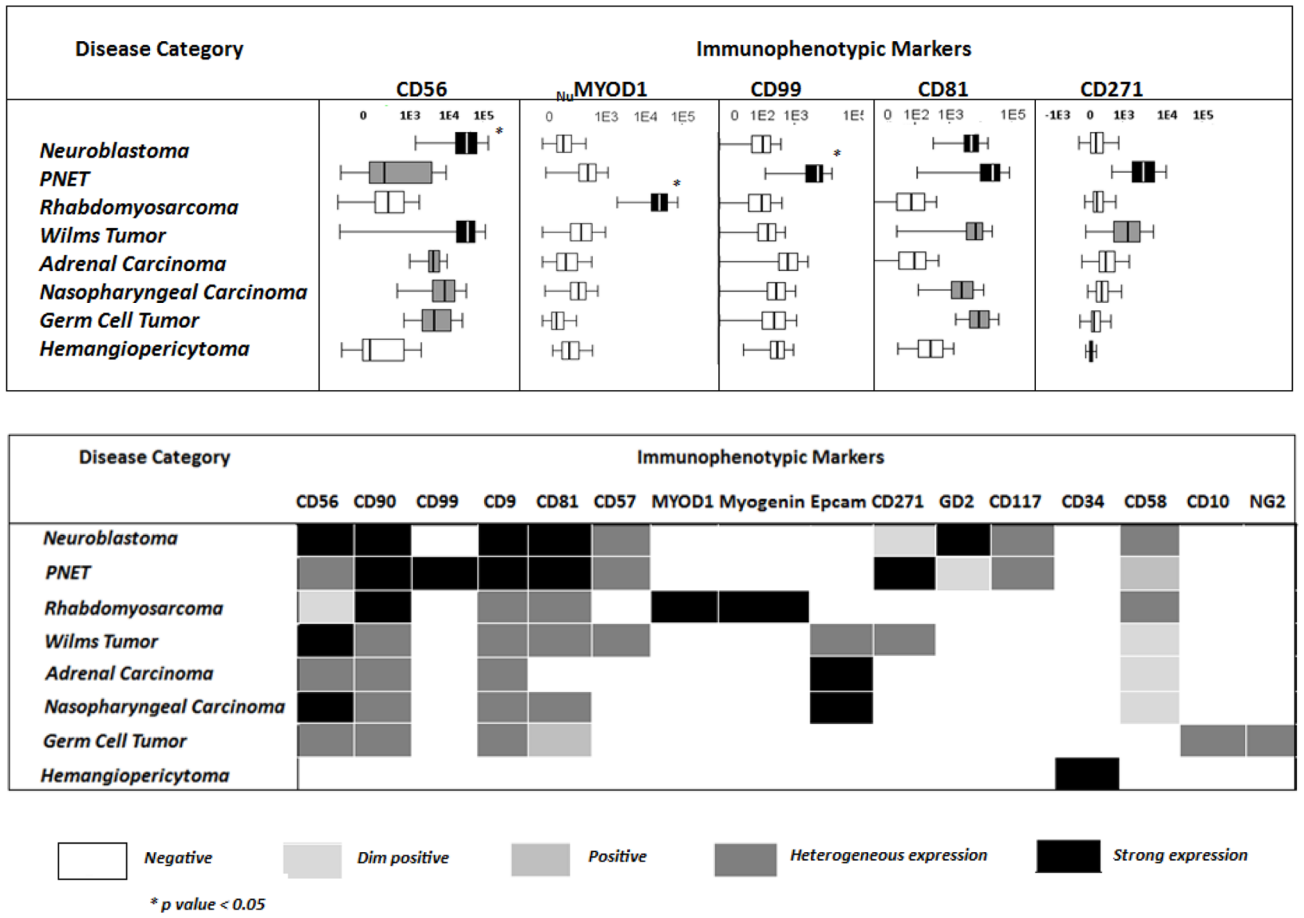
Pattern of expression of individual immunophenotypic markers in distinct diagnostic categories of pediatric solid tumors. Panel A: Heat map summarizing the intensity and pattern of expression of different markers in distinct diagnostic subtypes of pediatric solid tumors based on mean fluorescent intensity per/cell level. Panel B: Comparison of the mean fluorescent intensity expression of individual markers per/cell in different WHO subtypes of pediatric solid tumors. Boxes extend from the 25th to 75th percentiles, the lines in the middle represent median values while horizontal lines correspond to 95% confidence intervals.

Almost half of the non-hematopoietic tumors corresponded to neuroblastoma (12/26; 46%). They showed a uniform population of CD45^−^, CD56^+^, CD9^+^, CD81^hi^, GD2^+^ tumor cells with heterogeneous expression of CD57^−/+^ and CD58^+/++^; CD90 was positive in all but one tumor, whereas CD271 was partially expressed in only one tumor (Table 2). Interestingly, the only ganglioneuroblastoma tumor analyzed showed two clearly different populations of tumor cells: 39% had an immunophenotype identical to that of the other neuroblastomas, while the remaining cells (61%) displayed higher light scatter (FSC and SSC) and greater expression of CD56, CD81 and CD57. None of the other markers tested (e.g. CD99, CD38, CD19, CD20, _Cy_CD79a, CD34, _nu_myogenin, _nu_MYOD1) was expressed by neuroblastoma cells. From all tumors, neuroblastoma was the only GD2^+hi^ neoplasia, at the same time it also showed higher CD56 levels per cell (Table 2; Figures 1 and 2). Although PNET tumors showed a phenotype which resembled that of neuroblastoma (CD45^−^, CD56^hi^, CD90^+^, CD9^+^, CD81^+^, _nu_MYOD1^−^,_ nu_myogenin^−^ in the absence of B-, T- and myeloid-associated markers), they were negative for GD2, except for one with low expression on a small tumor population 48%, and showed stronger expression of CD99^hi^ and CD271^hi^ (Table 2; Figures 1 and 2).

**Table 2:** Pattern of antigen expression by tumor cells from different diagnostic categories of pediatric solid tumors.

WHO diagnosis	Immunophenotypic markers																			
	CD45	CD56	CD57	CD58	CD90	GD2	CD9	CD81	CD99	Myogenin	MYOD1	CD34	CD117	EpCAM	CD19	smCD3	CD4	CD10	CD38	CD271
**Neuroblastoma** (n = 12)*	−	+hi	+het	+het	+hi	+hi	+hi	+hi	−	−	−	−	+het	−	−	−	−	−	−	+lo
% of positive tumor cells		100±0	93±9	80±30	95±17	100	89±20	100±0					63±26							44±0§
N of positive cases		(12/12)	(8/12)	(3/12)	(11/12)	(12/12)	(12/12)	(12/12)					(8/12)							(1/12)
**PNET** (n = 2)	−	+het	+het	+	+hi	+lo	+hi	+hi	+hi	−	−	−	+het	−	−	−	−	−	−	+hi
% of positive tumor cells		97±5	54±0§	100±0	100±0	48±0	100	100	100				58±16							100±0
N of positive cases		(2/2)	(1/2)	(2/2)	(2/2)	(1/2)	(2/2)	(2/2)	(2/2)				(2/2)							(2/2)
**Rhabdomyosarcoma** (n = 4)	−	+lo	−	+het	+hi		+het	+het	+	+hi	+hi	−	−	−	−	−	−	−	−	−
% of positive tumor cells		69±37		67±0§	74±23		30±4	27±63	41±0§	100±0	100±0									
N of positive cases		(4/4)		(1/4)	(3/4)		(2/4)	(2/4)	(1/4)	(4/4)	(4/4)									
**Adrenal carcinoma** (n = 1)	−	+het	−	+lo	+het	−	+het	−	−	−	−	−	−	+hi	−	−	−	−	−	−
% of positive tumor cells		100		48	91		84							78						
**Nasopharyngeal carcinoma** (n = 1)	−	+hi	−	+lo	+	−	+	+	−	−	−	−	−	+hi	−	−	−	−	−	−
% of positive tumor cells		100		61	96		100	100						82						
**Germ cell tumor** (n = 3)	−	+het	−	−	+het	−	+het	+	−	−	−			−	−	−	−	+het	−	−
% of positive tumor cells		74±37			57±0§		67±0§	82±0§										63±0§		
N of positive cases		(2/3)			(1/3)		(1/3)	(1/3)										(1/3)		
**Hemangiopericytoma** (n = 1)	−	−	−	−	−	−	−	−	−	−	−	+hi	−	−	−	−	−	−	−	−
% of positive tumor cells												100								
**Wilms Tumor** (n = 2)	−	+hi	−	+lo	−	−	+het	+het	−	−	−	−	−	+	−	−	−	−	−	+het
% of positive tumor cells		100±0		100±0			93±10	94±9						22±2						97±0§
N of positive cases		(2/2)		(2/2)			(2/2)	(2/2)						(2/2)						(1/2)
**Burkitt lymphoma** (n = 3)	+	+	−	−	−	−	+	+	−	−	−	−	−	−	+hi	−	−	+hi	+hi	−
% of positive tumor cells	100±0	100±0§					78±31	100±0§							100±0			100±0	100±0	
N of positive cases	(3/3)	(1/3)					(2/3)	(1/3)							(3/3)			(3/3)	(3/3)	
**B**−**lymphoblastic lymphoma** (n = 2)	+lo	−	−	+	−	−	+	+	−	−	−	−	−	−	+hi	−	−	−	+hi	−
% of positive tumor cells	100±0			100±0			100±0	100±0							100±0				100±0	
N of positive cases	(2/2)			(2/2)			(2/2)	(2/2)							(2/2)				(2/2)	
**EBV-related T-lymphoma** (n = 2)	+	−	−	+	−	−	−	−	+	−	−	−	−	−	−	+	+	−	−	−
% of positive tumor cells	100±0			100±0					100±0							91±5	89±5			
N of positive cases	(2/2)			(2/2)					(2/2)							(2/2)	(2/2)			

−: negative; +lo :low expression levels/cells; +: positive; +hi: strong expression levels/cells.

Both CD7 and CD8 were systematically negative in all tumors analyzed.

* The only ganglioneuroblastoma tumor analyzed showed a similar profile but it contained two distinct populations which differed on CD56, CD9 and CD81 expression, in the absence of CD117.

CD271 was only partially present in one neuroblastoma tumor.

§ % of positive cells only among positive case.

Based on the same screening and characterization panels (panels 1 and 2 in Table S2, respectively), all four rhabdomyosarcomas (RMS) showed a specific _nu_MYOD1^hi^, _nu_myogenin^hi^ phenotype. Moreover, RMS cases were also CD45^−^, CD56^+lo^, CD90^+^ and CD57^−^; two expressed CD81^+^, CD9^+^ and CD58^+^ and one was partially positive for CD99 (40% of tumor cells ), a phenotypic pattern which was specific for this tumor subtype (Table 2; Figures 1 and 2).

The remaining 8 tumor samples corresponded to 5 distinct tumor subtypes (Wilms tumor, 2 cases; germ cell tumor, 3; adrenal carcinoma, nasopharyngeal carcinoma and hemangiopericytoma, one case each). Strong EpCAM expression was restricted to the two carcinomas, while hemangiopericytoma cells were the only displaying CD34^+hi^ expression; both groups of tumors systematically lacked CD45, B- and T-cell markers (Table 2; Figures 1 and 2). In turn, all germ cell tumors presented with a distinct CD45^−^, CD56^+^, CD10^+^, CD38^−^, CD19^−^, CD22^−^, NG2^+^ phenotype, except for CD10 and NG2 that were negative in 1/3 cases (Table 2 and Figure 2). Conversely, the two Wilms tumors showed two clearly distinct (coexisting) tumor cell populations with a common CD56^+^ and CD58^+^, CD45^−^, CD99^−^, GD2^−^, _nu_MYOD1^−^, _nu_myogenin^−^, CD10^−^ and NG2^−^ phenotype, but distinct reactivity (negative versus positive expression) for CD90, EpCAM and CD57 (Table 2; Figures 1 and 2).

Overall, these results show that distinct pediatric tumor subtypes were associated with unique and specific phenotypes. _Nu_MYOD1 and _nu_myogenin expression was restricted to RMS (Figure 1J; Figure2), CD99 was expressed at significant higher levels in PNET and a subpopulation of (embryonal) RMS (Figure 1I; Figure 2), whereas strong reactivity for GD2 was specific for neuroblastoma (Figure 1H; Figure 2), and a CD34^hi^ CD45^−^ phenotype was restricted to the hemangiopericytoma case studied (Figure 2). Other less specific markers such as CD56, CD9, CD57, CD81, CD99 also contributed to some differential diagnoses, e.g. the distinction between neuroblastoma and RMS (CD56,CD57,CD81) and between PNET and both RMS (CD9) and neuroblastoma (CD99, CD56, CD81 and CD57) (Figure 1I–J; Figure 2).

## Discussion

Pediatric cancer mainly derives from early lymphoid precursors and embryonic mesenchymal and neuroectodermal precursors, which may show similar morphological and histopathological patterns [3], [5]–[7]. Consequently, diagnosis of most pediatric tumors frequently requires further characterization of the neoplastic cells on e.g. immunophenotypical/immunocytochemical grounds. In turn, rapid diagnosis of such cases is crucial, since it directly influences the treatment decision-making process and patient outcome [2], [3]. Therefore, availability of fast techniques to precisely screen for the tumor cell lineage and establish the relevant differential diagnoses, are mostly welcome.

Until now, few studies have been reported which evaluate the utility of MFC immunophenotyping for the diagnostic screening and subclassification of pediatric solid tumors [11]–[13], [27]–[28], [31], [35]–[39], [41], [48]–[51] Noteworthy, a relatively low (59%) concordance rate between immunophenotyping of fine-needle aspirated specimens by flow-cytometry and conventional cytological analyses has been reported, due to low sample cellularity [36]. Interestingly, here we obtained enough viable cells in every sample analyzed by using mechanical disaggregation procedures of freshly-obtained and processed samples, supporting the notion that mechanical disaggregation keeps antigen expression together with an acceptable cell viability [16]–[18], [52].

Of note, none of the samples classified as reactive/inflammatory by cytological/histopatological criteria was misdiagnosed as cancer by MFC. In turn, all tumor specimens but two rather uncommon lymphoma samples, were identified as containing tumor by MFC. The two false negative cases observed could be due to the lack of specific markers for Reed-Stenberg and anaplastic lymphoma cells (e.g. CD30) in our screening panel (panel 1 in Table S2) and the relatively low frequency and/or viability of these cells in single cell suspensions. Prospective inclusion of additional markers (e.g. CD30) in the screening panel for the identification of these cells may potentially overcome this limitation [53], [54].

In pediatric patients, MFC has been mainly applied to the study of PB and BM samples from patients suspicious of suffering from acute leukemia. Despite this, early studies in neuroblastoma patients already showed BM infiltration by CD45^−^/CD56^+^ tumor cells, in the absence of proteins denoting hematopoietic commitment [29], [33]. Because of the relatively high frequency of metastatic BM involvement in neuroblastoma, this is by far the non-hematopoietic pediatric tumor mostly studied by MFC. Indeed, the phenotype of neuroblastoma cells has been recurrent evaluated in single or multicolor (3- or 4-color) antibody combinations both in BM and tumor tissue samples [35], [48]–[51], [55]. However, most of these studies were aimed at assessing minimal residual disease levels in the BM of neuroblastoma patients, without exploring the utility of immunophenotyping for the diagnostic screening and differential diagnosis of neuroblastoma *vs.* other pediatric tumors, as done here.

From all markers evaluated so far in pediatric solid tumors, CD56 (NCAM) has been most frequently investigated [31]–[34]. Interestingly, CD56 is expressed by most pediatric solid tumors [37]–[39], [51], as also found in our cases. However, the amount of CD56 expression largely varied among different tumor subtypes. Neuroblastoma and PNET were those tumors which displayed the highest CD56 levels, supporting the utility of CD56 both to discriminate non-hematopoietic vs. hematopoietic neoplasms [31], [34], and for the differential diagnosis between distinct subtypes of CD45^−^ non-hematopoietic pediatric solid tumors. Similarly, several studies have evaluated the utility of CD81 and CD9 (together with CD45^−^ and CD56^+^), for the discrimination between neuroblastoma cells and BM hematopoietic cells, for staging purposes [13], [48]–[49], [55], without extending such analysis to other subtypes of pediatric solid tumors. Among our cases, CD81 and CD9 showed a variable and heterogeneous pattern of expression with limited utility as individual markers, in the differential diagnosis between neuroblastoma and other pediatric tumors. However, once combined with other molecules, CD81 contributed to the discrimination of neuroblastoma and PNET. Despite this, GD2 and CD271 were the two most useful markers to differentiate between neuroblastoma (GD2^+hi^ CD271^−/lo^) and PNET (GD2^−/lo^ CD271^+hi^). Overall, these results support the notion that CD271^hi^ expression identified on PNET may be associated with the mesenchymal stem cell origin of these tumors [56]. Interestingly, in the only neuroblastoma patient that showed CD271^−/+lo^ tumor cells, CD271 expression was restricted to the primary tumor, while negative in metastatic BM cells; this could potentially be due to a different degree of tumor cell maturation at both sites, absence of CD271 being associated with a more immature and aggressive tumor behavior [57]–[58]. Of note, CD99 was also highly-expressed in PNET, while typically negative in the other tumors, suggesting that in addition to CD271, CD99 may also contribute to the diagnosis of PNET.

Few MFC immunophenotypic studies of RMS have been reported so far. In line with our observations such studies showed that RMS cells typically display a CD45^−^, CD56^+^, CD90^+^, _nu_myogenin^+^ phenotype with variable expression of CD57, desmin, vimentin and CD99 [31], [36], [39], [41]. Unfortunately, in these studies, these and other markers (e.g. _nu_MYOD1) have not been systematically investigated in other non-RMS tumors. In line with previous immunohistochemical studies [40], [59], in our series both_ nu_myogenin and _nu_MYOD1 emerged as specific for RMS cells.

Differential diagnosis of pediatric small round cell tumors (SRCT) with respect to both Wilms tumor and non-Hodgkin lymphoma, frequently remains a challenge. Here, we clearly show that tumor cells from these later two entities can be clearly discriminated from other SRCT on immunophenotypic grounds. Thus, Wilms tumor was the only pediatric cancer subtype which consisted of two coexisting tumor cell populations, with distinct immunophenotypes: CD45^−^ CD56^+^ EpCAM^+^ CD90^−^ *vs.* CD45^−^ CD56^+^ CD90^+^ EpCAM^−^. These observations are in line with the reported coexistence of epithelial (e.g. EpCAM^+^, CD90^−^) and mesenchymal (EpCAM^−^, CD90^+^) cell components in Wilms tumors by histopathology [60]–[61]. In turn, childhood carcinomas are extremely rare neoplasms whose immunophenotype has not been previously characterized by MFC. Here we showed high expression of EpCAM, CD56, CD58 and CD90 by flow cytometry in two pediatric carcinomas (an adrenal carcinoma and a nasopharyngeal carcinoma). Since all other non-carcinoma tumor cell samples (except one subset of tumor cells in the Wilms tumors) were negative for EpCAM, this marker could also be potentially useful for the diagnosis of this subtype of pediatric tumors, particularly if combined with CD90.

In summary, our results indicate that MFC is a useful complementary tool for fast diagnostic screening and classification of pediatric cancer. Combined assessment of CD45, CD56, CD81, CD99, EpCAM, GD2, _nu_MYOD, _nu_myogenin and CD271, together with other specific B-cell (e.g. CD19) and T-cell (e.g. _Cy_CD3) markers, emerges as providing particularly useful information for such purpose.

## Supporting Information

Table S1 Results expressed as number of patients or samples and ^*^median values and range between brackets. NA: not applicable. ^**^Among the non-neoplastic samples, 9 reactive/inflammatory samples and 8 non-infiltrated samples from patients with cancer in another localization, were analyzed. ^♣^In 6 cases, a tumor mass plus sample(s) from another site were simultaneously analyzed (tumor mass plus BM in 2 cases; tumor mass plus urine in 2 cases; tumor mass plus ascitic fluid in one case; tumor mass plus BM and PB, one case). In five cases, the primary site was not accessed because the risk of the procedure and only BM was analyzed. ^♠^In one case, tumor masses plus BM and PB were simultaneouslyanalyzed. In two cases the primary site was not accessed because the risk of the procedure, and only BM was analyzed. ^∞^In one case, a cervical lymph node plus PB were simultaneouslyanalyzed.^ ♪^In two cases, a tumor mass plus BM were simultaneously analyzed.
(DOC)

Table S2 For each Antibody, the marker/CD marker (clone and commercial source) are displayed. BD: Becton-Dickinson Biosciences (San José, CA, USA). Beckman Coulter (Brea, CA, USA) CA, USA). Cytognos Cytognos (Salamanca, Spain). Dako (Glostrup, Denmark). Invitrogen (Carlsbad, CA, USA). Exbio (Prague, Czech Republic) Miltenyi Biotec (Cologne, Germany) e Biolegend,CA, USA) *EuroFlow (San Diego,CA,USA) *EuroFlow ALOT tube [45].
(DOC)

Table S3 Results are expressed as mean percentage of cells ±one standard deviation and range between brackets. ^*^One sample with inflammatory bowel disease had a subpopulation of 32% CD45^−^/CD56^−^/Epcam^+hi^ identified as normal/residual epithelial cells. The expression of EpCAM in these cells was much stronger than that found in carcinoma cells with a pattern resembling that of normal epithelial cells.
(DOC)

Table S4 Results expressed as median (range) percentage of cells from the whole sample cellularity, except for those groups for which only one case was studied^*^One sample was from a lymph node infiltrated by neuroblastoma cells **^§^** One sample was a face tumor with massive infiltration by inflammatory cells.
(DOC)
