# Supplementary material for: Contribution of Multiparameter Flow Cytometry Immunophenotyping to the Diagnostic Screening and Classification of Pediatric Cancer
Source: PLoS One. 2013 Mar 5;8(3):e55534. doi: 10.1371/journal.pone.0055534 (PMC3589426; doi:10.1371/journal.pone.0055534)
Supplement: Table S1 — Results expressed as number of patients or samples and *median values and range between brackets. NA: not applicable. **Among the non-neoplastic samples, 9 reactive/inflammatory samples and 8 non-infiltrated samples from patients with cancer in another localization, were analyzed. ♣In 6 cases, a tumor mass plus sample(s) from another site were simultaneously analyzed (tumor mass plus BM in 2 cases; tumor mass plus urine in 2 cases; tumor mass plus ascitic fluid in one case; tumor mass plus BM and PB, one case). In five cases, the primary site was not accessed because the risk of the procedure and only BM was analyzed. ♠In one case, tumor masses plus BM and PB were simultaneouslyanalyzed. In two cases the primary site was not accessed because the risk of the procedure, and only BM was analyzed. ∞In one case, a cervical lymph node plus PB were simultaneouslyanalyzed. ♪In two cases, a tumor mass plus BM were simultaneously analyzed. (DOC) [file pone.0055534.s001.doc]

**Supplementary table 1.**General information about the patients (n=40) and samples (n=52) analyzed

|  | Patients | Samples |
| --- | --- | --- |
|  | (n=40) | (n=52) |
| **Age at diagnosis^*^** | 5 years | NA |
|  | (1-14 years) |  |
| **Gender:** |  | NA |
| Male | 21 (52.5%) |  |
| Female | 19 (47.5%) |  |
| **Diagnosis:** |  |  |
| Neoplastic Diseases | 31 | 35 |
| - Localized | 14 | 22 |
| - Metastatic | 17 | 13 |
| Non-neoplastic diseases | 9 | 17^**^ |
| **Localization of tumor mass** |  |  |
| Abdomen/pelvis | 22 | 17^^ |
| Thoracic | 7 | 5^^ |
| Cervical lymph node | 7 | 7^∞^ |
| Face | 3 | 3^♪^ |
| Soft parts | 1 | 1 |
| **Time of analysis** |  |  |
| At diagnosis | 28 | 39 |
| On chemotherapy | 4 | 4 |
| Other | 9 | 9 |

Results expressed as number of patients or samples and ^*^median values and range between brackets. NA: not applicable.^**^Among the non-neoplastic samples, 9 reactive/inflammatory samples and 8 non-infiltrated samples from patients with cancer in another localization, were analyzed. ^^In 6 cases, a tumor mass plus sample(s) from another site were simultaneously analyzed (tumor mass plus BM in 2 cases; tumor mass plus urine in 2 cases; tumor mass plus ascitic fluid in one case; tumor mass plus BM and PB, one case). In five cases, the primary site was not accessed because the risk of the procedure and only BM was analyzed.^^ In one case, tumor masses plus BM and PB were simultaneouslyanalyzed. In two cases the primary site was not accessed because the risk of the procedure, and only BM was analyzed.^∞^In one case, a cervical lymph node plus PB were simultaneouslyanalyzed. ^♪^In two cases, a tumor mass plus BM were simultaneouslyanalyzed.
