# Supplementary material for: Contribution of Multiparameter Flow Cytometry Immunophenotyping to the Diagnostic Screening and Classification of Pediatric Cancer
Source: PLoS One. 2013 Mar 5;8(3):e55534. doi: 10.1371/journal.pone.0055534 (PMC3589426; doi:10.1371/journal.pone.0055534)
Supplement: Table S2 — For each Antibody, the marker/CD marker (clone and commercial source) are displayed. BD: Becton-Dickinson Biosciences (San José, CA, USA). Beckman Coulter (Brea, CA, USA) CA, USA). Cytognos Cytognos (Salamanca, Spain). Dako (Glostrup, Denmark). Invitrogen (Carlsbad, CA, USA). Exbio (Prague, Czech Republic) Miltenyi Biotec (Cologne, Germany) e Biolegend,CA, USA) *EuroFlow (San Diego,CA,USA) *EuroFlow ALOT tube [45]. (DOC) [file pone.0055534.s002.doc]

S**upplementary Table 2. Panels of monoclonal antibodies (MAbs) used in the present study for the diagnostic screening of pediatric cancer (panel 1: orientation panel) and further characterization of hematopoietic (panels 3-5: lymphoma panels) and non-hematopoietic tumors (panel 2: solid tumor panel).**

| **Panel 1: Orientation panel for the diagnostic screening phase** | | | | | | | | | | | |
| --- | --- | --- | --- | --- | --- | --- | --- | --- | --- | --- | --- |
| **Fluorochome** | **PacB** | **PacO** | **FITC** | **PE** | **PePerCPCy5.5** | **PECy7** | | | **APC** | **APC-H7** | |
| **Tube 1*** | **cyCD3** | **CD45** | **MPO** | **cyCD79a** | **CD34** | **CD19** | | | **CD7** | **sCD3** | |
|  | UCHT1 | HI30 | MPO-7 | HM57 | 8G12 | J3119BeckmanCoulter | | | 124-1D1 | SK7 | |
|  | BDBiosciences | Invitrogen | Dako | Dako | BD Biosciences |  | | | eBioscinece | BD Biosciences | |
| **Tube 2** | **CD20** | **CD45** | **CD8/sIgλ** | **CD56/sIgκ** | **CD19/CD4** | **CD56** | | | **CD3** |  |  |
|  | 2H7 | HI30 | Lymphoclonal | Lymphoclonal | Lymphoclonal | N901/NKH1 | | | Lymphoclonal |  |  |
|  | eBioscience | Invitrogen | Cytognos | Cytognos | Cytognos | Beckman Coulter | | | Cytognos |  |  |
| **Panel 2: Characterization panel for samples suspected of containing pediatric solid tumor cells** | | | | | | | | | | | |
| **Tube 1** |  | **CD45** | **CD57** | **CD90** | **CD34** | **CD56** | | | **Epcam** | |  |
|  |  | HI30 | HNK-1 | 5.00E+10 | 8G12 | N901/NKH1 | | | EBA-1 | |  |
|  |  | Invitrogen | BD Biosciences | BD Biosciences | BD Biosciences | Beckman Coulter | | | BD Biosciences | |  |
| **Tube 2** | **CD20** | **CD45** | **CD99** | **CD81** | **CD9** | **CD56** | | | **CD117** | |  |
|  | 2H7 | HI30 | TÜ12 | JS-81 | M-L13 | N901/NKH1 | | | 104D2 | |  |
|  | eBioscience | Invitrogen | BD Biosciences | BD Biosciences | BD Biosciences | Beckman Coulter | | | BD Biosciences | |  |
| **Tube 3** |  | **CD45** | **CD58** | **CD38** |  | **CD56** | | | **CD10** | |  |
|  |  | HI30 | 1C3 | HB-7 |  | N901/NKH1 | | | HI10A | |  |
|  |  | Invitrogen | BDBioscience | BD Biosciences |  | Beckman Coulter | | | BD Biosciences | |  |
| **Tube 4** |  | **CD45** |  | **CD271** |  | **CD56** | | |  |  |  |
|  |  | HI30 |  | C40-1457 |  | N901/NKH1 | | |  |  |  |
|  |  | Invitrogen |  | BD Biosciences |  | Beckman Coulter | | |  |  |  |
|  | **and 4 single tubes with the following antibodies + rabbit anti-IgG FITC:** | | | | | | | | | | |
| **Tubes 5-8** |  |  | **_Nu_MYOD** | **_Nu_Myogenin** | **GD2** | **_Cy_Desmina** |  |  | |  |  |
|  |  |  | Moab 5.8ª | F5D | 14.G2a | RD301 |  |  | |  |  |
|  |  |  | BD Biosciences | BD Biosciences | BDBiosciences | BD Biosciences |  |  | |  |  |

| **Panel 3: Characterization panel for samples suspected of containing pediatric B-cell lymphoma cells** | | | | | | | | | | |
| --- | --- | --- | --- | --- | --- | --- | --- | --- | --- | --- |
| **Tube 1** |  | **CD45** | **_Nu_Tdt** |  | **CD34** | **CD19** | | **CD22** |  |  |
|  |  | HI30 | HT6 |  | 8G12 | J3119 | | S-HCL-1 |  |  |
|  |  | Invitrogen | Dako |  | BD Biosciences | BeckmanCoulter | | BD Biosciences |  |  |
| **Tube 2** | **CD20** | **CD45** | **_Cy_Bcl2** | **CD38** | **CD34** | **CD19** | | **CD10** |  |  |
|  | 2H7 | HI30 | 124 | HB-7 | 8G12 | J3119 | | HI10A |  |  |
|  | eBioscience | Invitrogen | Dako | BD Biosciences | BD Biosciences | BeckmanCoulter | | BD Biosciences |  |  |
| **Tube 3** |  | **CD45** | **_Cy_IgM** |  | **CD34** | **CD19** | |  |  |  |
|  |  | HI30 | polyclonal rabbit serum |  | 8G12 | J3119 | |  |  |  |
|  |  |  | Dako |  |  |  | |  |  |  |
|  |  | Invitrogen |  |  | BD Biosciences | BeckmanCoulter | |  |  |  |
| **Tube 4** | **CD21** | **CD45** | **CD15/CD65** | **NG2** | **CD34** | **CD19** | | **CD123** |  |  |
|  | LT-21 | HI30 | MMA/88H7 | 124 | 8G12 | J3119 | | AC145 |  |  |
|  | Exbio | Invitrogen | BD Biosciences/ | BeckmanCoulter | BD Biosciences | BeckmanCoulter | | MiltenyiBiotec |  |  |
|  |  |  | Beckman Coulter |  |  |  | |  |  |  |
| **Panel 4: Characterization panel for samples suspected of containing pediatric T-cell lymphoma cells** | | | | | | | | | | |
| **Tube 1** | **_Cy_CD3** | **CD45** | **_Nu_Tdt** | **CD56** | **CD4** | **CD8** | **CD7** | | **_Sm_CD3** | |
|  | UCHT1 | HI30 | HT6 | C5.9 | 8K3 | SFCI21Thy2D3 | 124-1D1 | | SK7 | |
|  | BD Biosciences | Invitrogen | Dako | Cytognos | BDBiosciences | Beckman Coulter | eBioscinece | | BD Biosciences | |
|  |  |  |  |  |  |  |  | |  | |
| **Tube 2** | **_Cy_CD3** | **CD45** | **_Nu_Tdt** | **CD99** | **CD5** |  | **CD1a** | | **_Sm_CD3** | |
|  | UCHT1 | HI30 | HT6 | TÜ12 | L17F12 |  | HI149 | | SK7 | |
|  | BD Biosciences | Invitrogen | Dako | BD Biosciences | BDBiosciences |  | BDBiosciences | | BD Biosciences | |
| **Tube 3** | **_Cy_CD3** | **CD45** | **CD2** | **CD117** | **CD4** | **CD8** | **CD7** | | **_Sm_CD3** | |
|  | UCHT1 | HI30 | RPA-2.10 | 104D2 | 8K3 | SFCI21Thy2D3 | 124-1D1 | | SK7 | |
|  | BD Biosciences | Invitrogen | BDBioscience | BDBioscience | BDBiosciences | Beckman Coulter | eBioscinece | | BD Biosciences | |
| **Tube 4** | **HLA-DR** | **CD45** |  | **CD13** | **CD4** | **CD8** | **CD123** | | **_Sm_CD3** | |
|  | L243 | HI30 |  | L138 | 8K3 | SFCI21Thy2D3 | AC145 | | SK7 | |
|  | Biolegend | Invitrogen |  | BDBioscience | BDBiosciences | Beckman Coulter | MiltenyiBiotec | | BD Biosciences | |
| **Panel 5: Characterization panel for samples suspected of containing Hodgkin lymphoma and anaplastic lymphoma cells** | | | | | | | | | | |
| **Tube 1** | **CD20** | **CD45** | **CD15** | **CD30** | **CD5** | **CD56** | **CD10** | |  |  |
|  | 2H7 | HI30 | MMA | BerH8 | **L17F12** | N901/NKH1 | HI10A | |  |  |
|  | eBioscience | Invitrogen | BD Biosciences | BD Biosciences | **BD Biosciences** | Beckman Coulter | BD Biosciences | |  |  |

For each For each Antibody, the marker/CD marker (clone and commercial source) are displayed. BD: Becton-Dickinson Biosciences (San José, CA, USA). Beckman Coulter (Brea, CA, USA) CA, USA). Cytognos Cytognos (Salamanca, Spain). Dako (Glostrup, Denmark). Invitrogen (Carlsbad, CA, USA). Exbio (Prague, Czech Republic) Miltenyi Biotec (Cologne, Germany) e Biolegend ,CA, USA) *EuroFlow ( San Diego,CA,USA) *EuroFlow ALOT tube [45].
