# Supplementary material for: Contribution of Multiparameter Flow Cytometry Immunophenotyping to the Diagnostic Screening and Classification of Pediatric Cancer
Source: PLoS One. 2013 Mar 5;8(3):e55534. doi: 10.1371/journal.pone.0055534 (PMC3589426; doi:10.1371/journal.pone.0055534)
Supplement: Table S3 — Results are expressed as mean percentage of cells ±one standard deviation and range between brackets. *One sample with inflammatory bowel disease had a subpopulation of 32% CD45−/CD56−/Epcam+hi identified as normal/residual epithelial cells. The expression of EpCAM in these cells was much stronger than that found in carcinoma cells with a pattern resembling that of normal epithelial cells. (DOC) [file pone.0055534.s003.doc]

**Supplementary table 3. Distribution of different cell populations in inflammatory/ reactive samples (n=9) corresponding to cervical lymph nodes (n=6) and 3 abdominal and soft tissue masses (n=3)**

| **Cell subsets** | **T-lymphocytes** | | | | **B-lymphocytes** | | **Neutrophils** |
| --- | --- | --- | --- | --- | --- | --- | --- |
| **Type of samples** | ***CD3^+^ T-cells*** | ***CD4^+^/CD8 – CD8^+^/ CD4^-^*** | ***CD4^-^/CD8 –*** | ***CD4^+^/CD8 ^+^*** | ***CD19^+^CD20^+^ B-cells*** | ***sIgκ ^+^sIgλ^+^*** |  |
|  |  | ***T-cells T-cells*** | ***T-cells*** | ***T-cells*** |  | ***B-cells B-cells*** |  |
| **Cervicallymph nodes** |  |  |  |  |  |  |  |
|  | ***64% ±20%*** | ***44% ±19% 15% ±8%*** | ***3.4% ±3%*** | ***0.5% ±0.7%*** | ***34%±20%*** | ***19%±12% 16%±9%*** | **1.5% ±1.5%** |
|  | ***(44%-93%)*** | ***(26%-81%) (6%-30%)*** | ***(1%-8%)*** | ***(0%-1.7%)*** | ***(5.4%-56%)*** | ***(3%-32%) (2.3%-24%)*** | **(0%-4.4%)** |
|  |  |  |  |  |  |  |  |
| **Abdominal and soft tissue masses**^*^ | ***61% ±11%*** | ***35% ±17% 20% ±10%*** | ***2.6% ±4.4%*** | ***2% ±2.6%*** | **6.6% ±4%** | ***3.6% ±2.2% 3% ±1.6%*** | **21% ±23%** |
|  | ***(51%-73%)*** | ***(21%-54%) (14%-31%)*** | ***(0%-13%)*** | ***(0%-5.1%)*** | **(3%-11%)** | ***(1.4%-6%) (1.5%-4.7%)*** | **(1.1%-46%)** |

Results are expressed as mean percentage of cells ±one standard deviation and range between brackets.^*^One sample with inflammatory bowel disease had a subpopulation of 32% CD45^-^/CD56^-^/Epcam^+hi^ identified as normal/residual epithelial cells. The expression of EpCAM in these cells was much stronger than that found in carcinoma cells with a pattern resembling that of normal epithelial cells
