# Supplementary material for: Contribution of Multiparameter Flow Cytometry Immunophenotyping to the Diagnostic Screening and Classification of Pediatric Cancer
Source: PLoS One. 2013 Mar 5;8(3):e55534. doi: 10.1371/journal.pone.0055534 (PMC3589426; doi:10.1371/journal.pone.0055534)
Supplement: Table S4 — Results expressed as median (range) percentage of cells from the whole sample cellularity, except for those groups for which only one case was studied*One sample was from a lymph node infiltrated by neuroblastoma cells § One sample was a face tumor with massive infiltration by inflammatory cells. (DOC) [file pone.0055534.s004.doc]

**Supplementary table 4. Distribution of different populations of residual normal/reactive hematopoietic and non-hematopoietic cells in samples from non-hematopoietic solid tumors (n=14)**

| **Hystopathological Diagnosis** | **Total T-cells** | **CD4^+^-T cells** | **CD8^+^-T cells** | **CD4^-^/CD8**⁻ **Tcells** | **CD4^+^/CD8 ^+^ T-cells** | **B-Cells** | **Neutrophils** | **Endothelial cells** | **Mesenchymal cells** |
| --- | --- | --- | --- | --- | --- | --- | --- | --- | --- |
|  |  |  |  |  |  |  |  | **(CD45^-^/CD34^+^)** | **(CD45/CD90^+^/CD56⁻)** |
| **Neuroblastoma (5/12)** | 14.6(7.4-59**^*^**) | 48(40-64) | 29(26.5-45.5) | 14.5(6.3-19) | 0.7(0-6.7) | 5.9(2.3-16.5) | 2.8(0.5-51**^§^**) | 1(0.1-1.3) | 0.8(0.5-3) |
|  |  |  |  |  |  |  |  |  |  |
|  |  |  |  |  |  |  |  |  |  |
| **Ganglioneuroblastoma (1/1)** | 4.2 | 64 | 28 | 6.2 | 0.4 | 0.2 | 0.8 | - | 7.8 |
|  |  |  |  |  |  |  |  |  |  |
|  |  |  |  |  |  |  |  |  |  |
| **PNET (2/2)** | 5.6(3.7-7.6) | 31.5(18-45) | 62.2(45.4-79) | 6.3(3-9.6) | 0.15(0-0.3) | 0.5(0.3-0.7) | 6.9(0.8-13) | 0.15(0.1-0.2) | 0.1 |
|  |  |  |  |  |  |  |  |  |  |
|  |  |  |  |  |  |  |  |  |  |
| **Wilms Tumors (2/2)** | 2(1.3-2.7) | 29(14-44) | 60(51.2-69) | 17 | - | 0.4 | 0.5(0.1-1) | 0.3(0.1-0.6) | 0.6(0.1-1.2) |
|  |  |  |  |  |  |  |  |  |  |
|  |  |  |  |  |  |  |  |  |  |
| **Adrenal Carcinoma (1/1)** | 5 | 33 | 59 | 8.3 | - | 0.2 | 3 | - | - |
|  |  |  |  |  |  |  |  |  |  |
|  |  |  |  |  |  |  |  |  |  |
| **Nasopharyngeal Carcinoma (1/1)** | 11 | 54 | 46 | - | - | 7 | 4.6 | 0.4 | 4.4 |
|  |  |  |  |  |  |  |  |  |  |
| **Germ Cell Tumor (2/2)** | 9.3(7.7-11) | 51(47-55) | 39(35-43) | 10 | 0 | 0.4(0.2-0.7) | 0.8 | 0 | 0.1 |

Results expressed as median (range) percentage of cells from the whole sample cellularity, except for those groups for which only one case was studied. **^*^**One sample was from a lymph node infiltrated by neuroblastoma cells **^§^** One sample was a face tumor with massive infiltration by inflammatory cells.
